# What Will Our Answer Be?

**Published:** 1896-08

**Authors:** 


### WHAT WILL OUR ANSWER BE?

FRANCE, in April last, notified this country that it would not permit imports of cattle to that country without subjecting them to the tuberculin-test. That government has also provided for the sanitary inspection of all cattle owned and kept in France. The action of France, Germany, and Italy, in adopting measures to protect their flocks from tuberculosis, will no doubt be followed by Great Britain and other countries. Should the latter nation take similar steps, it will mean an additional restriction upon the exportation of cattle from this country, and few of our States, particularly our great western reserve, are in position to defend themselves from this restriction. True it is that those sections of our country are literally free from tuberculosis among the cattle, but this will scarcely give them protection from such proposed measures. Those who have looked upon the means adopted in Massachusetts, Maine, New York, Connecticut, and other States as oppressive and too radical, will soon realize that they must follow the same course. We do not want other countries to send us more tuberculosis than we have, and they are perfectly justified in not wishing any greater amount to deal with than they have at home. We have no disposition to question the wisdom of these measures in other countries, and America must meet them by unquestioned evidence that we are sending them cattle free from tuberculosis, just as we can assure them of our freedom from contagious pleuro-pneumonia.

